# Weighted straight skeletons in the plane^☆^

**DOI:** 10.1016/j.comgeo.2014.08.006

**Published:** 2015-02

**Authors:** Therese Biedl, Martin Held, Stefan Huber, Dominik Kaaser, Peter Palfrader

**Affiliations:** a David R. Cheriton School of Computer Science, University of Waterloo, Waterloo, Ontario N2L 1A2, Canada; b Universität Salzburg, FB Computerwissenschaften, 5020 Salzburg, Austria; c Institute of Science and Technology Austria, 3400 Klosterneuburg, Austria

**Keywords:** Straight skeleton, Generalization, Characterization, Ambiguity, Positive and negative weights

## Abstract

We investigate weighted straight skeletons from a geometric, graph-theoretical, and combinatorial point of view. We start with a thorough definition and shed light on some ambiguity issues in the procedural definition. We investigate the geometry, combinatorics, and topology of faces and the roof model, and we discuss in which cases a weighted straight skeleton is connected. Finally, we show that the weighted straight skeleton of even a simple polygon may be non-planar and may contain cycles, and we discuss under which restrictions on the weights and/or the input polygon the weighted straight skeleton still behaves similar to its unweighted counterpart. In particular, we obtain a non-procedural description and a linear-time construction algorithm for the straight skeleton of strictly convex polygons with arbitrary weights.

## 1 Introduction

The straight-skeleton S(P) of a simple polygon *P* is a skeleton structure that was introduced to computational geometry by Aichholzer et al. [1] about 20 years ago. Its definition is based on a wavefront propagation process where the polygon's edges move inwards at unit speed. The straight skeleton, roughly speaking, is the skeleton structure that results from the interference patterns of the wavefront edges. Aichholzer and Aurenhammer [2] later generalized the definition to planar straight-line graphs. Since their introduction, a lot of applications appeared in different research areas, and multiple algorithms to compute the straight skeleton have been introduced [3].

Eppstein and Erickson [4] were the first to mention the *weighted straight skeleton* where the wavefront edges may move with arbitrary but fixed speeds. They claim that their algorithm to compute the unweighted straight skeleton in O(n85+ϵ) time and space also works, without major changes, for weighted straight skeletons. Weighted straight skeletons have many applications: Barequet et al. [5] use weighted straight skeletons in order to define the initial wavefront topology for straight skeletons of polyhedra. Haunert and Sester [6] use the weighted straight skeleton for topology-preserving area collapsing in geographic maps. Laycock and Day [7] and Kelly and Wonka [8] use weighted straight skeletons to model realistic roofs of houses. Aurenhammer [9] investigated fixed-share decompositions of convex polygons using weighted straight skeletons with specific positive weights. Sugihara [10] employs weighted straight skeletons for the interactive design of pop-up cards.

Although algorithms, applications, and even simple implementations [11] of weighted straight skeletons are known, only limited research has been conducted on the weighted straight skeleton itself. The only known results are that the simple definition based on wavefront propagation may lead to ambiguities [3], [8] and that the lower envelope characterization by Eppstein and Erickson [4] does not apply. In this paper, we carefully define weighted straight skeletons, shed light on the ambiguity in the procedural definition, investigate geometric, graph-theoretical and combinatorial properties of weighted straight skeletons, and compare those with properties of unweighted straight skeletons. In particular, we show that weighted straight skeletons of simple polygons may have cycles and crossings. Furthermore, we investigate necessary conditions for the weights or the polygon such that the weighted straight skeleton of a simple polygon is a planar tree.

## 2 Preliminaries

The definition of the straight skeleton S(P) of a simple polygon *P* is based on a so-called wavefront propagation of *P* where all edges of *P* move inwards in parallel and at unit speed. (This definition is readily extended to polygons with holes.) The wavefront, denoted by $W_{P}(t)$, has the shape of a mitered offset curve of *P* for small *t*. As *t* increases, $W_{P}$ changes its topology. Such changes are called events and we can distinguish between two main types: An *edge event* happens when an edge *e* collapses to zero length and vanishes. A *split event* happens when a reflex wavefront vertex *v* reaches another part of the wavefront and splits it into parts. Typically, *v* meets a wavefront edge whose split causes the entire wavefront to split into two parts. However, if *v* meets one or more other wavefront vertices, then more complicated splits of the wavefront into multiple parts are possible. Either event causes local changes in the topology of the wavefront so that the resulting wavefront again consists of a collection of simple polygons.

**Fig. 1:**
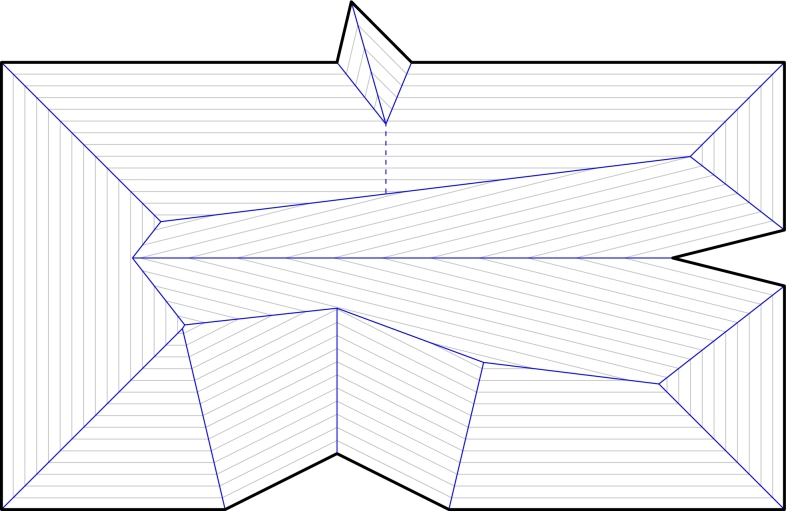
The straight skeleton S(P) of the input polygon *P* (bold) is defined by wavefronts emanated from *P*. The dashed arc is traced by a ghost vertex.

The straight skeleton S(P) is defined as the set of loci traced out by the vertices of $W_{P}(t)$ for all t≥0, see Fig. 1. Additionally, some loci are added to the straight skeleton in case of parallel edges as follows: (a) If two parallel edges *e* and $e^{\prime }$ that move in opposite directions become overlapping during an event, then the region common to *e* and $e^{\prime }$ is added to the straight skeleton, while the region(s) that belongs to exactly one of them remains in the wavefront. (b) If two parallel edges *e* and $e^{\prime }$ that move in the same direction become adjacent due to an edge event, then their common endpoint is considered a vertex of the wavefront. We call this a *ghost vertex*. This vertex moves perpendicular to *e* and $e^{\prime }$.^1^

Each wavefront vertex traces out an *arc* of S(P). Since wavefront vertices move along bisectors of edges of *P*, the arcs of S(P) are straight-line segments. Every event of $W_{P}$ corresponds to a locus where arcs of S(P) meet and give rise to a *node* of S(P). See Fig. 1 for an example. The straight skeleton S(P) is interpreted as a graph, and one can show that it is a tree [1]. Also, no two arcs of the straight skeleton cross since the wavefront moves inwards towards the unswept region. Hence, S(P)∪P is a planar straight-line graph. The inner faces of S(P)∪P are called straight-skeleton *faces*.

Let *e* be an edge of a polygon *P*, and let the *wavefront fragments of e at time t* be the union of segments of $W_{P}(t)$ that originated from *e*. We denote this set by e(t), and it may comprise none, one, or many segments, depending on whether *e* participated in edge events and/or split events. We consider segments in e(t) to be *open* line segments. Every straight-skeleton face f(e) is traced out by (the fragments of) one wavefront edge *e*, thus $f(e):={⋃}_{t>0}e(t)$. It can be shown that f(e) is monotone with respect to the supporting line of *e* [1], [2], and its lower chain is convex [3]. Furthermore, the boundary of each face f(e) corresponds to a cycle in P∪S(P).

### 2.1 Roof models

Aichholzer et al. [1] introduced the *roof model*, which is a handy way of interpreting the straight skeleton. One considers the wavefront propagation embedded in three-space where the *z*-axis constitutes time. Then $W_{P}$ traces out a surface, namely the roof model $T(P):={⋃}_{t\geq 0}W_{P}(t)\times \{t\}$. This concept gives us the means to investigate $W_{P}$ over its entire lifespan. Note that we can obtain S(P) from T(P) by projecting the edges of T(P) onto the plane $R^{2}\times \{0\}$. Conversely, we can obtain T(P) from S(P) by lifting all nodes of S(P) by their orthogonal distance to the respective input edges of *P*. These distances correspond to the times when the nodes were swept by the wavefront.

The roof model is sometimes also called *terrain model* since T(P) is a *terrain*, i.e., any line parallel to the *z*-axis intersects it in at most one point. As this property may be violated for the weighted version of straight skeletons, we prefer the term “roof model”.

### 2.2 Weighted straight skeletons

The weighted straight skeleton differs from the (unweighted) straight skeleton only in the speed σ(e)∈R∖{0} with which an edge *e* moves in the wavefront. We call *σ* the *weight function* and σ(e) the *weight* of *e*. The wavefront moves such that the fragments e(t) of *e* at time *t* are on the line $\bar{e}+t\cdot \sigma (e)\cdot n(e)$, where $\bar{e}$ is the supporting line of *e*, and n(e) is the inward unit normal of *e*. For σ(e)<0, an edge *e* moves outward with speed |σ(e)|.^2^

The definitions of edge event, $W_{P}(t)$, S(P), e(t), f(e), and T(P) for the unweighted straight skeleton carry over verbatim to the weighted straight skeleton. We use an additional parameter *σ* in $W_{P}(t,\sigma )$, S(P,σ), and T(P,σ) in order to refer to their weighted counterparts. The major difference, however, lies in the richness of the notation of a split event: A split event happens when the wavefront becomes non-simple because a wavefront vertex *v* meets another part of the wavefront. The vertex *v* no longer needs to be reflex. Also, a split event does not necessarily split the wavefront into multiple parts. Even worse, different possibilities may exist on how to proceed with the wavefront propagation such that the wavefront still remains a planar collection of simple polygons between events. We discuss this in more detail in Section 4.

Negative weights may cause wavefront components to propagate towards infinity. The corresponding roof model is unbounded in this case. From a combinatorial point of view, we add a node at infinity for every wavefront component *C* that still exists at a sufficiently large time such that all events already have been processed. Each such node is incident to the infinite arcs that are traced out by vertices of *C*. Doing so gives us the important property that the boundary of each face f(e) is a cycle in P∪S(P,σ) even for unbounded faces.

While most definitions carry over, it is not at all clear which of the properties of the straight skeleton, such as planarity, connectedness, acyclicity, or monotonicity of faces, remain valid in the weighted version. This is the main topic of this paper and will be discussed in Section 5.

## 3 Velocity of wavefront vertices

For later argumentations, we first develop a formula that describes how the movement of a wavefront vertex *v* is determined by the propagation of the two incident wavefront edges $e_{1}$ and $e_{2}$ which we assume to be not collinear. (See Section 4 for a discussion of degeneracies.) We use $\sigma (v)\in R^{2}$ for the velocity vector of *v*.

**Fig. 2:**
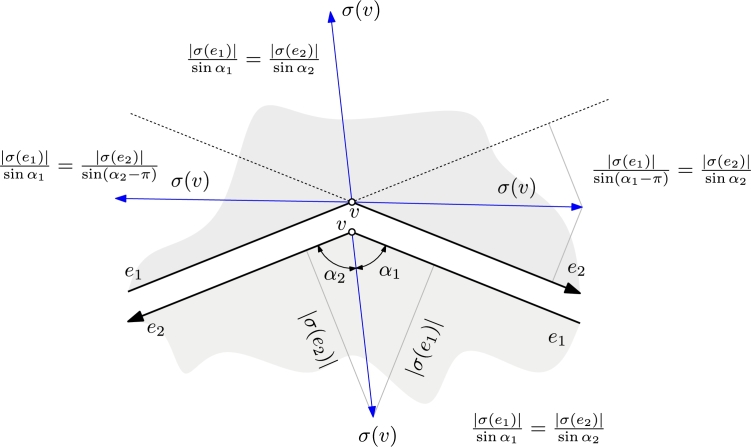
The velocity vector *σ*(*v*) of a wavefront vertex *v* with left edge $e_{1}$ and right edge $e_{2}$ lies in one of four sectors spanned by the supporting lines of $e_{1}$ and $e_{2}$.

Notice that σ(v) lies in one of the four sectors spanned by the supporting lines of $e_{1}$ and $e_{2}$, and we know which one of these to use from the signs of both $\sigma (e_{1})$ and $\sigma (e_{2})$. Therefore, we can determine which edge among $e_{1},e_{2}$ is the first edge in counter-clockwise direction from σ(v) and call this the *left edge* of *v*. Similarly, we determine the first edge in clockwise direction and call this *right edge* of *v*. After possible renaming, assume that $e_{1}$ is the left edge and $e_{2}$ is the right edge as shown in Fig. 2. We denote by $\alpha_{1}$ the counter-clockwise angle between σ(v) and $e_{1}$ and by $\alpha_{2}$ the clockwise angle between σ(v) and $e_{2}$.

We have multiple cases to consider, based on the signs of $\sigma (e_{1})$ and $\sigma (e_{2})$ and on whether *v* is convex or reflex. With our choice of naming for $\alpha_{1}$ and $\alpha_{2}$ dependent on the wedge containing σ(v), these cases can be combined, and we can express ‖σ(v)‖ by $\left|\sigma (e_{i})\right|$ and $\alpha_{i}$ as shown in Fig. 2 as follows:

(1) $$\left\|\sigma (v)\right\|=\left\{ \begin{array}{cc} \frac{\left|\sigma (e_{i})\right|}{\sin\alpha_{i}} & \text{for}\;\alpha_{i}<\pi \\ \frac{\left|\sigma (e_{i})\right|}{\sin(\alpha_{i}-\pi )} & \text{for}\;\alpha_{i}>\pi \end{array}\right.,$$

for all i∈{1,2}. Note that $\alpha_{i}=\pi$ is impossible as $\sigma (e_{i})\neq 0$. In case that $\alpha_{i}>\pi$, we have $\mathrm{sign}(\sigma (e_{1}))\neq \mathrm{sign}(\sigma (e_{2}))$. Using $\sin(\alpha_{i}-\pi )=-\sin\alpha_{i}$, it follows that in all cases

(2) $$\frac{\sigma (e_{1})}{\sin\alpha_{1}}=\frac{\sigma (e_{2})}{\sin\alpha_{2}}.$$

Denoting by $\gamma =\alpha_{1}+\alpha_{2}$ the angle spanned by $e_{1}$ and $e_{2}$ on the side where *v* moves to, we see that

$$\frac{\sin\alpha_{1}}{\sigma (e_{1})}=\frac{\sin(\gamma -\alpha_{1})}{\sigma (e_{2})}=\frac{\sin\gamma \cos\alpha_{1}-\cos\gamma \sin\alpha_{1}}{\sigma (e_{2})},$$

and hence we obtain

(3) $$\cot\alpha_{1}=\frac{\cos\gamma +\frac{\sigma (e_{2})}{\sigma (e_{1})}}{\sin\gamma }.$$

From the case distinctions on $\mathrm{sign}(\sigma (e_{1}))$ and $\mathrm{sign}(\sigma (e_{2}))$ we can uniquely determine $\alpha_{1}$ and, using Eq. (1), obtain ‖σ(v)‖.

## 4 Ambiguity of definition

### 4.1 Ambiguity at edge events

**Fig. 3:**
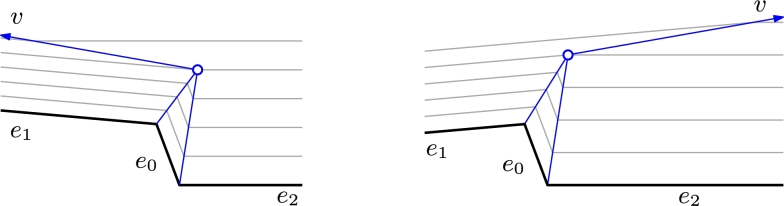
The definition of straight skeletons is ambiguous when two parallel wavefront edges with different weights become adjacent.

Presume that we have an edge event where edge $e_{0}$ disappears, leaving its adjacent edges $e_{1}$ and $e_{2}$ to become adjacent with common endpoint *v* as shown in Fig. 3.

Recall that Eq. (3) describes the angle at which vertex *v* moves after the event. However, the right-hand side of this equation is indeterminate for sin⁡(γ)=0, i.e., when $e_{1}$ and $e_{2}$ are parallel. Unfortunately, as *γ* monotonically approaches *π*, we may obtain two different limit cases:

(4) $$\lim_{\gamma ↗\pi }\alpha_{1}=0\;\lim_{\gamma ↘\pi }\alpha_{1}=\pi .$$

Therefore, and as already alluded to by Kelly and Wonka [8] and Huber [3], the definition of the weighted straight skeleton is ambiguous whenever parallel wavefront edges with different weights become adjacent. In the case where $\sigma (e_{1})=\sigma (e_{2})$, we can apply de l'Hôpital's rule to obtain $\lim_{\gamma \to \pi }\cot\alpha_{1}=0$, and hence $\alpha_{1}=\pi /2$. Similarly, $\alpha_{1}=\pi \pm \pi /2$ if $\sigma (e_{1})=-\sigma (e_{2})$ and γ→0. But in all other cases, there is no unique definition of a straight skeleton.

Whenever two edges become adjacent in the ambiguous case, there are different options on how to proceed. At first sight, it appears possible to treat this case equal to the well-defined case and keep propagating both edges. Unfortunately, regardless of which direction we choose for the bisector of these two edges, we will run into a problem: the difference in speed of edge propagation results in an ever growing gap between the two parts of the wavefront made up by $e_{1}$ and $e_{2}$. This gap could be filled by creating a new wavefront edge that again connects $e_{1}$ and $e_{2}$. However, this approach seems inadvisable: This new wavefront edge would not belong to any specific input edge and it would not necessarily be parallel to any input edge either. Also, depending on the exact definition of this new edge, most likely it would have a propagation speed of zero, resulting in a degenerate face.

We suggest that the only reasonable way of handling the ambiguity is to terminate propagation of one of the edges in favor of the other. The limit cases suggested by Eq. (4) support this approach as they set *α* to either 0 or *π*. It is unclear which of the two limits one should choose as both choices appear equally justified. To get a deterministic algorithm, one canonical strategy might be to always pick the faster, or always the slower, of the two edges to continue its propagation. The first choice emphasizes the idea of the faster wavefront edge overtaking the slower one and leads to the nice property that the arc traced out by *v* is convex in the roof model. The second choice increases the slope of the roof and, thus, the volume under the roof.

Let the left edge $e_{1}$ be the one we decided to stop. We add $e_{1}$ to S(P). Then, edge $e_{2}$ is enlarged to cover the wavefront previously belonging to $e_{1}$ and continues its propagation at its defined speed.

This ambiguity can already arise at time t=0, i.e., for the input polygon: Consider a polygon with two consecutive edges that are collinear. Unless the weights of these two edges are identical, we end up with one face of S(P) that has zero area.

**Fig. 4:**
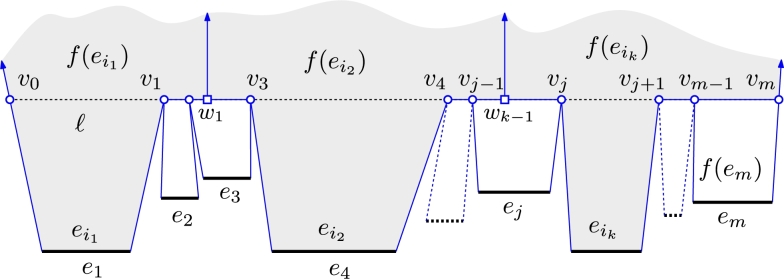
Continuing the wavefront propagation if multiple parallel wavefront edges become adjacent when simultaneously reaching the line *ℓ*.

Note that the situation can become even more complicated if multiple parallel wavefront edges with different weights become consecutive, as depicted in Fig. 4. Like in the simple case, there are many equally justified ways to continue the wavefront propagation. For the sake of simplicity, we will describe just one of them.

Assume, as in Fig. 4, that at some point in time a line *ℓ* contains part of the wavefront. Let a maximal contiguous part of the wavefront on *ℓ* consist of vertices and edges $v_{0},e_{1},v_{1},\ldots ,e_{m},v_{m}$ (in order). Also assume for now that $e_{1},\ldots ,e_{m}$ all approach *ℓ* from the same half-plane. Let $1\leq i_{1}<\cdots <i_{j}<\cdots <i_{k}\leq m$ be indices such that edges $e_{i_{1}},\ldots ,e_{i_{k}}$ are those edges with maximal weight, i.e., $\sigma (e_{i_{1}})=\cdots =\sigma (e_{i_{k}})=\max_{1\leq i\leq m}\sigma (e_{i})$. We now modify the wavefront such that those edges take over the wavefront while all other edges disappear. For each j=1,…,k−1, we create a ghost vertex $w_{j}$ halfway between $v_{i_{j}}$ and $v_{i_{j+1}-1}$, i.e., we add ghost vertices between all edges of maximal speed. Then, we extend each of the edges $e_{i_{1}},\ldots ,e_{i_{k}}$ on both sides to their respective ghost vertices (or to $v_{0}$ and $v_{m}$ at the ends) and drop all other edges. This replaces the wavefront between $v_{0}$ and $v_{m}$ with edges $\left(v_{0},w_{1}\right)$, $\left(w_{1},w_{2}),\ldots ,(w_{k-2},w_{k-1}),(w_{k-1},v_{m}\right)$. Thus, these edges take over this part of the wavefront from all other edges and continue to propagate while the remaining edges disappear.

If not all edges on *ℓ* approach from the same half-plane, then any non-empty line segment that is common to two edges in opposite direction becomes an arc of the weighted straight skeleton. We otherwise proceed as above: The edge(s) with the maximum speed take over all other adjacent edges of the wavefront that reside on *ℓ*.

### 4.2 Ambiguities at coinciding split events

A second ambiguity may arise when multiple split events happen at the same time and at the same locus *p* of the wavefront. How should the topology of the wavefront change? It should be done in such a way that afterwards the wavefront again consists of a planar collection of simple polygons, i.e., so that the wavefront near *p* has no crossing after the event.

**Fig. 5:**
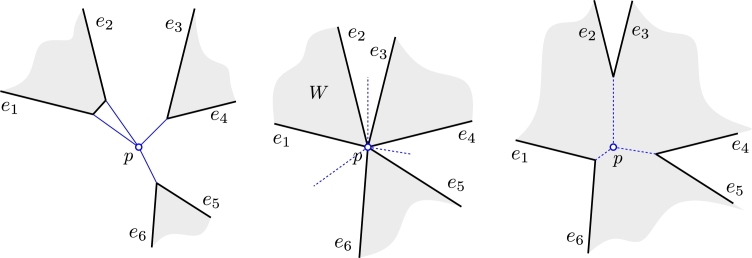
Three wavefront vertices meet simultaneously at *p*. Left: Before the event the pairing of wavefront edges is $\left(e_{1},e_{2}),(e_{3},e_{4}),(e_{5},e_{6}\right)$. Middle: At the event, the edges tessellate the neighborhood of *p* into interior-disjoint wedges. Every second wedge was already swept by the wavefront. Right: After the event the pairing changes to $\left(e_{2},e_{3}),(e_{4},e_{5}),(e_{6},e_{1}\right)$.

Denote by $e_{1},\ldots ,e_{2k}$ the edges that end at *p* when the event happens, see Fig. 5. We say $e_{i}$ and $e_{j}$ are *paired* if the wavefront near *p* consisted of edge $e_{i}$, then (for the pairing before the event) possibly a few edges that vanish during the event at *p*, and then edge $e_{j}$. For a specific pairing before the event, we are interested in a valid pairing after the event such that the wavefront remains crossing-free.

Roughly speaking, if $e_{1},\ldots ,e_{2k}$ are sorted clockwise around *p* and the pairing before the event was $\left(e_{1},e_{2}),(e_{3},e_{4}),\ldots ,(e_{2k-1},e_{2k}\right)$, then we define the pairing $\left(e_{2},e_{3}),(e_{4},e_{5}),\ldots ,(e_{2k},e_{1}\right)$ as the *standard resolution*, see Fig. 5.

Lemma 1 *For positive weights the standard resolution always gives a valid pairing and, moreover, it is the only valid solution.*

Proof We first remark that for positive weights a locus is never swept twice by the wavefront, as we show in Section 5.1. Furthermore, the wavefront is planar until it reaches *p*. The essential observation is the following: When the wavefront reaches *p*, the neighborhood of *p* is tessellated into interior-disjoint wedges bounded by $e_{1},\ldots ,e_{2k}$, and every other wedge is completely swept by the wavefront. Assume that $e_{1},\ldots ,e_{2k}$ are sorted clockwise around *p* and the wedge *W* between $e_{1},e_{2}$ is already swept. That means that before the event the edges $e_{1}$ and $e_{2}$ resided in *W*. As the wedges are interior-disjoint, $e_{1}$ and $e_{2}$ were paired before the event. By analogous arguments for the other edges, $\left(e_{1},e_{2}),(e_{3},e_{4}),\ldots ,(e_{2k-1},e_{2k}\right)$ was the pairing before the event.

The standard resolution alters the pairing to $\left(e_{2},e_{3}),(e_{4},e_{5}),\ldots ,(e_{2k},e_{1}\right)$. Observe that all edge pairs $e_{2i}$ and $e_{2(i\;\mathrm{mod}\;k)+1}$, with 1≤i≤k, reside in different unswept wedges, which proves the validity of the standard resolution. Assume now that there is an alternative pairing. We may assume that $e_{2}$ is paired with an edge other than $e_{3}$. But then $e_{2}$ would necessarily cross an already swept area which is impossible for positive weights.  □

**Fig. 6:**
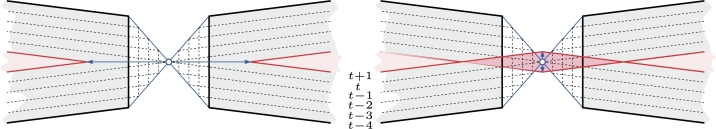
Two edge events (for the vertical edges) and multiple split events coincide at time *t*. We must retain the pairing as it was after vanishing edges have been excised; otherwise crossings result.

For negative weights, it is not at all obvious why a pairing without crossings should even exist. Fig. 6 shows an example where the standard pairing can *not* be applied, as it results in crossings.

We can transform the problem of finding a suitable pairing to a stable roommates problem, as defined by the order of intersections along the edges. This stable roommate problem can be shown to possess a stable matching. However, the details of this technique and the proofs are non-trivial and will be covered in a separate paper. (A preliminary version appeared as Biedl et al. [12].)

Another difficulty is that there may be multiple valid pairings that have no crossings.

Lemma 2 *There exists an instance of coinciding events, with weights in* {+1,−1} *, for which multiple post-event wavefronts without crossings exist.*

**Fig. 7:**
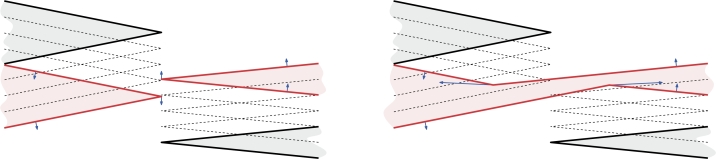
When multiple reflex vertices meet at an event, there may be multiple possible new wavefronts. Different wavefronts after the event are shown in the figures on the left and on the right. The old pairing (left) and the standard pairing (right) both yield simple wavefronts.

Proof The example is shown in Fig. 7.  □

## 5 Properties of straight skeletons

For the sake of simplicity and clarity, we assume in the following that the ambiguities discussed in Section 4 do not occur. That means we assume that (i) there are no two parallel edges with different weights that become consecutive due to an edge event or that are consecutive initially, and (ii) that two or more events do not happen simultaneously at the same place unless they are all edge events. The latter implies that split events are exclusively caused by vertices reaching the interior of an edge of the wavefront. But, as we show now, even under these assumptions many seemingly natural properties do not hold for weighted straight skeletons. Table 1 summarizes all results.

**Table 1:** Results for a polygon with *h* holes, a simple polygon, and a convex polygon, with *n* denoting the number of vertices of the polygon *P* and *v* denoting the number of nodes of S(P).

Polygon with holes	σ≡1		*σ* positive		*σ* arbitrary	

S(P) is connected	*✓*	Lemma 15	*✓*	Lemma 15	×	Lemma 12
S(P) has no crossing	*✓*	Lemma 6	*✓*	Lemma 6	×	Lemma 3
*f*(*e*) is monotone w.r.t. *e*	*✓*	as in [1]	×	Lemma 11	×	Lemma 11
bd *f*(*e*) is a simple polygon	*✓*	as in [1]	×	Lemma 8	×	Lemma 8
T(P,σ) is *z*-monotone	*✓*	Lemma 4	*✓*	Lemma 4	×	Lemma 5
S(P) has *n* + *v* − 1 + *h* arcs	*✓*	Corollary 17	*✓*	Corollary 17	×	Lemma 3
S(P) is a tree	×	Corollary 17	×	Corollary 17	×	Lemma 12


Simple polygon	σ≡1		*σ* positive		*σ* arbitrary	

S(P) is connected	*✓*	[1]	*✓*	Lemma 15	*✓*	Corollary 14
S(P) has no crossing	*✓*	[1]	*✓*	Lemma 6	×	Lemma 3
*f*(*e*) is monotone w.r.t. *e*	*✓*	[1]	×	Lemma 11	×	Lemma 11
bd *f*(*e*) is a simple polygon	*✓*	[1]	*✓*	Lemma 9	×	Lemma 10
T(P,σ) is *z*-monotone	*✓*	[1]	*✓*	Lemma 4	×	Lemma 5
S(P) has *n* + *v* − 1 arcs	*✓*	[1]	*✓*	Corollary 17	×	Lemma 3
S(P) is a tree	*✓*	[1]	*✓*	Corollary 19	×	Lemma 3


Convex polygon	σ≡1		*σ* positive		*σ* arbitrary	

S(P) is connected	*✓*	[1]	*✓*	Lemma 15	*✓*	Corollary 14
S(P) has no crossing	*✓*	[1]	*✓*	Lemma 6	×	Lemma 20
*f*(*e*) is monotone w.r.t. *e*	*✓*	[1]	*✓*	Corollary 25	*✓*	Corollary 25
bd *f*(*e*) is a simple polygon	*✓*	[1]	*✓*	Lemma 9	*✓*	Corollary 25
T(P,σ) is *z*-monotone	*✓*	[1]	*✓*	Lemma 4	*✓*	Theorem 24
S(P) has *n* + *v* − 1 arcs	*✓*	[1]	*✓*	Corollary 17	*✓*	Corollary 29
S(P) is a tree	*✓*	[1]	*✓*	Corollary 19	*✓*	Lemma 28

Lemma 3 *There exists a simple polygon P with weights chosen from the set* {+1,−1} *such that* S(P,σ) *has crossings and cycles.*

**Fig. 8:**
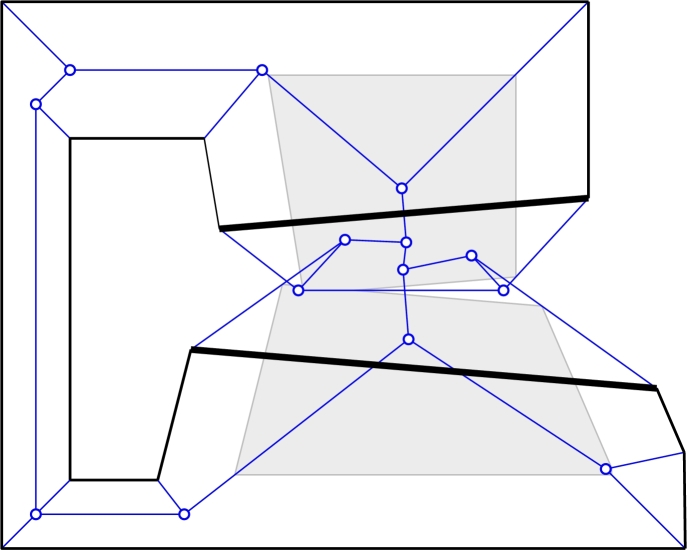
S(P,σ) of a simple polygon *P* may have crossings and cycles. A wavefront at some time *t* is shown in gray. All edges have weight +1, except the two bold edges, which have a negative weight of −1.

Proof The polygon is shown in Fig. 8.  □

### 5.1 Terrains and crossings

We now want to show that if the weights are positive, then the straight skeleton has no crossing. For this (and other claims later) it will help to study when the roof model T(P,σ) is a terrain.

Lemma 4 *Let P be a polygon (possibly with holes). If* σ(e)>0 *for all wavefront edges e, then the roof model* T(P,σ) *is a terrain and its z-projection equals P.*

Proof Since all weights are positive, the wavefront edges emanated by *P* move towards the interior of *P*. Therefore, after each event of $W_{P,\sigma }$, the trajectories of the newly born wavefront vertices point to the area within *P* that has not yet been swept by the wavefront. Thus, no wavefront vertex can ever reach a locus that has already been swept—such a vertex would have met another part of the wavefront before and the vertex would have been annihilated. Accordingly, the wavefront $W_{P,\sigma }$ stays within *P* and no locus of *P* is swept more than once by the wavefront. On the other hand, each locus of *P* is swept at least once, since otherwise the boundary of the unswept region would be the wavefront, and therefore not yet empty. □

Note that Lemma 4 fails to hold as soon as there is a negative weight as the following lemma shows.

Lemma 5 *There exists a simple polygon P with weights chosen from the set* {+1,−1} *such that* T(P,σ) *is not a terrain.*

Proof The example shown in Fig. 8 contains loci that are swept more than once. Hence, T(P,σ) is not a terrain. In fact, it can be modified easily such that some loci are swept an arbitrary number of times.  □

Lemma 6 *Let P be a polygon (possibly with holes). If* σ(e)>0 *for all wavefront edges e, then* S(P,σ) *has no crossings.*

Proof This holds by Lemma 4 since the locus *p* of any crossing must have been covered at least twice by the wavefront. But then the line parallel to the *z*-axis through *p* would intersect T(P,σ) twice.  □

We provide one more result, showing that, while weighted straight skeletons lack many of the properties of unweighted straight skeletons, their terrains are, nevertheless, not entirely arbitrary. The next lemma generalizes a theorem by Aichholzer et al. [1] that states that rain does not cumulate on roofs stemming from straight skeletons; instead raindrops can run off. In this sense, we define a *pit* of T(P,σ) as a point *p* on T(P,σ) for which no *z*-monotone path from *p* to *P* on T(P,σ) exists.

Lemma 7 *Let P be a polygon (possibly with holes) and let σ be an arbitrary weight function. Then* T(P,σ) *has no pit.*

Proof Consider a point *p* on the roof and say it was reached at time t>0. Let *p* lie in the closure of the face f(e) swept by wavefront fragments emanated from the input edge *e*. This face was traced out during the wavefront propagation process, i.e., there is a (not necessarily strictly) *t*-monotone path from *e* to *p*. Thus, in the roof, there is a *z*-monotone path ascending from *e* at z=0 to *p*. Therefore, no point *p* of the roof can be a pit.  □

### 5.2 Straight-skeleton faces

We later want to argue that under some assumptions the straight skeleton is connected. To do so, we first study some properties of the faces of edges. Recall that $f(e)={⋃}_{t>0}e(t)$, where e(t) are the open line segments that result from edge *e* at time *t*. Clearly, f(e) is connected (no fragment of *e* suddenly appears during a wavefront process) and its boundary bdf(e) consists of arcs of the straight skeleton and the input edge *e*.

Lemma 8 *There exists a polygon P with holes and with positive weights chosen from the set* {1,3} *such that* bdf(e) *is not a simple polygon.*

**Fig. 9:**
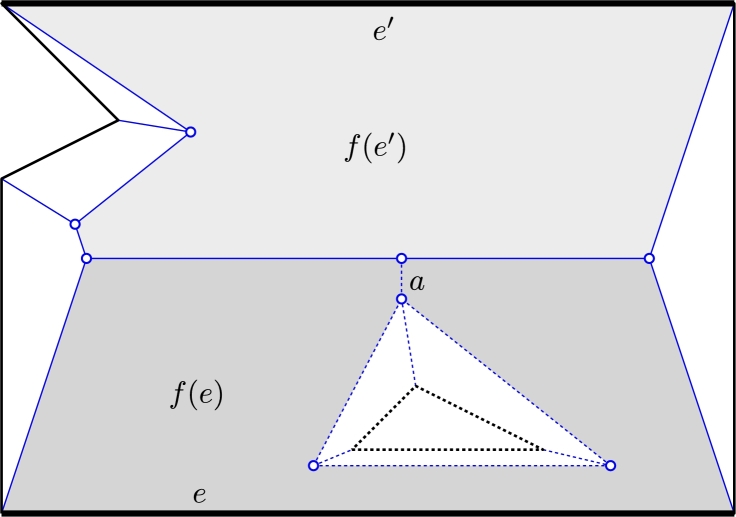
A polygon with hole may have a non-simple face. A simple polygon may have a non-monotone face. The dotted features are caused by the hole. The bold edges have weight 3, the others have weight 1.

Proof Polygon *P* is shown in Fig. 9 (include the dotted features), with edge *e* being the bottom-most horizontal edge. During the wavefront process, edge *e* gets split when it meets the hole. But since *e* moves faster than the edges of the hole, two fragments of *e* later re-combine. According to our definition of straight skeleton, a ghost vertex is created that traces the arc *a* in Fig. 9. The boundary of f(e), viewed as a polygon, is not simple and can be interpreted to contain the arc *a* twice. (Even without this arc, the boundary bdf(e) would not be one simple polygon.)  □

Lemma 8 used a polygon with holes. Whether for simple polygons a similar situation can arise depends on whether the weights are all positive or not.

Lemma 9 *Let P be a simple polygon and let σ be an assignment of positive weights to the edges of P. Then* bdf(e) *is a simple polygon for all edges e.*

Proof Note that f(e) is an open, simply-connected set: Whenever a fragment splits, the pieces never re-merge due to the ghost vertices. Hence bdf(e) is a weakly-simple^3^ polygon, and the only way that it could be not simple is by having a point *p* that is incident to three or more edges of the boundary of f(e); see Fig. 9 around *a*. This can only happen if two fragments $s_{1},s_{2}$ in e(t) become adjacent in the wavefront. By tracing from $s_{1}$ and $s_{2}$ back to *e* while residing inside f(e), we can find a closed Jordan curve *C* inside clf(e) that encloses a point outside clf(e).

For positive weights the roof model T(P) projects to *P* (Lemma 4), so C⊂clf(e)⊂P. Since *P* is simple, any point inside *C* also belongs to *P*. Since some point inside *C* does not belong to clf(e), some other face $f(e^{\prime })$ is inside *C*. But by planarity (Lemma 6) the edge $e^{\prime }$ of this face is inside *C* as well. Since *C* contains no edges of *P*, edge $e^{\prime }$ cannot be connected to the edges at the exterior face of *P*. So *P* has a hole, a contradiction.  □

Lemma 10 *There exists a simple polygon P with weights chosen from the set* {−1,+1,+3} *such that* bdf(e) *is not a simple polygon for an edge e of P.*

**Fig. 10:**
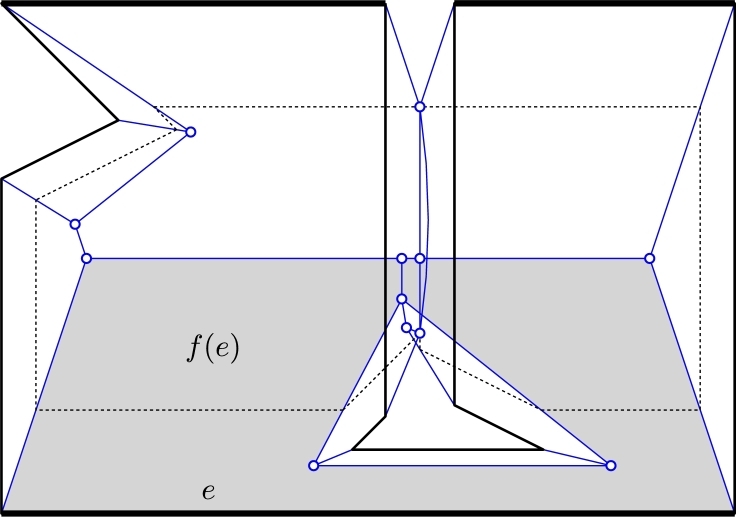
A simple polygon where face *f*(*e*) (shaded) has a non-simple boundary. One wavefront is depicted by dotted lines. The bold edges have weight 3 and the two vertical edges that form the corridor have weight −1. All other edges have weight 1. The arc between the two corridor edges geometrically coincides with other arcs.

Proof The polygon is shown in Fig. 10.  □

If all weights are the same, then bdf(e) is simple even for a polygon with holes, because f(e) is monotone with respect to the supporting line of *e* [1]. We note here that f(e) need not be monotone if we allow different weights. This is already obvious from Fig. 9, Fig. 10 but may happen even for simple polygons and positive weights.

Lemma 11 *There exists a simple polygon P with weights chosen from the set* {1,3} *such that for one edge e the face* f(e) *is not monotone with respect to the supporting line of e.*

Proof The face $f(e^{\prime })$ in Fig. 9, with the dotted features omitted, is not monotone.  □

### 5.3 Connectivity

The unweighted straight skeleton S(P) of a polygon *P* with holes is always connected. In fact, S(P) is even of the same homotopy type as *P*. The weighted straight skeleton, however, need not even be connected.

Lemma 12 *There exists a polygon P with holes and an assignment σ of weights to edges such that* S(P,σ) *is not connected.*

Proof If all weights are negative, then no straight skeleton arcs exist in the interior of *P*. However, straight skeleton arcs do exist in each component of $R^{2}\setminus P$. Thus, S(P,σ) is not connected.  □

The following lemma serves as a tool to prove connectedness. The lemma basically says that straight-skeleton features that are connected via the wavefront at any time are also connected within the final straight skeleton.

Lemma 13 *Let* $S_{t}(P,\sigma )$ *denote the straight-skeleton features traced by* $W_{P,\sigma }$ *until time t. If two points* $p,q\in S_{t}(P,\sigma )$ *are path-connected on* $S_{t}(P,\sigma )\cup W_{P,\sigma }(t)$ *, then they are path-connected on* S(P,σ) *.*

Proof Assume that *p* and *q* are two path-connected points on $S_{t}(P,\sigma )\cup W_{P,\sigma }(t)$. We prove this lemma by induction on the events of $W_{P,\sigma }$ in chronological order. We need to check that connectivity between *p* and *q* is maintained despite the changes of $W_{P,\sigma }$ caused by events.

An event may simply remove a collapsed edge of $W_{P,\sigma }(t)$ (edge event), remove a collapsed component of $W_{P,\sigma }(t)$ (edge event), split a component of $W_{P,\sigma }(t)$ (split event), or merge components (split event). In any case, a straight-skeleton node *v* is created to which arcs are incident that were traced by the vertices of each involved component of the wavefront. However, even if the wavefront is split into multiple components, each component remains connected to *v* by at least one arc that is traced by a vertex of each component. That is, an event never disconnects a component from the node that is created by the event.^4^

By induction, *p* and *q* are thus path-connected within $S_{t^{\prime }}(P,\sigma )\cup W_{P,\sigma }(t^{\prime })$ for $t^{\prime }$ after the last event. All remaining wavefront components move towards infinity. As we add an infinite node for each escaping wavefront component, S(P,σ) can be considered to result from $S_{t^{\prime }}(P,\sigma )\cup W_{P,\sigma }(t^{\prime })$ by contracting each component of $W_{P,\sigma }(t^{\prime })$ to a node. Hence, *p* and *q* are also path-connected within S(P,σ).  □

Corollary 14 S(P,σ) *is connected for simple polygons.*

Proof This holds even for negative weights as the initial wavefront of *P* consists of only one connected component.  □

Lemma 15 S(P,σ) *is connected for polygons with holes P and positive weights.*

Proof Consider the wavefront emanating from some hole. At some point, it either merges with some other wavefront during a split event; then the claim holds by induction (and Corollary 14) since $W_{P,\sigma }$ then has fewer components. Or it collapses during an edge event. But this is impossible, since for positive weights the wavefront of the hole moves towards the inside of *P* and hence encloses increasingly more area.  □

### 5.4 Bounds on the number of arcs

It is well-known that the unweighted straight skeleton of a simple polygon is a tree [1]. As we will see, for positive weights the weighted straight skeleton is also a tree. We show an even stronger statement, which bounds the number of arcs even in the presence of holes.

In the following, we will use that P∪T(P,σ) is a 2-manifold in order to apply the formula of Euler–Poincaré. As we mentioned at the beginning of Section 5, every event is either an edge event or a split event that was caused by a wavefront vertex meeting the interior of a wavefront edge. It is easy to see that a local neighborhood at each of the corresponding vertices of T(P,σ) is topologically equivalent to the plane. (Fig. 6 shows an example where the corresponding vertex causes T(P,σ) not to be a 2-manifold. Note that this example does not meet our requirements as two events coincide.) Also recall that we insert an infinite straight-skeleton node for every wavefront polygon that propagates towards infinity. Topologically, we can interpret this as follows: For a sufficiently large time such that all events have already been processed, we topologically glue together the points of *C* for every remaining wavefront polygon *C*. Hence, a neighborhood of infinite vertices is again topologically equivalent to the plane.

Lemma 16 *Let P be a polygon with n vertices and h holes and let σ be an assignment of arbitrary weights to the edges of P. Assume the body enclosed by* P∪T(P,σ) *has genus g. Denoting the number of straight-skeleton nodes and arcs by v and e, it holds that*

e=n+v−1+2g−h.

Proof We consider the body enclosed by P∪T(P,σ). Recall that all faces of T(P,σ) are weakly-simple polygons since they have been traced out by edges of the wavefront over time and we add ghost vertices whenever fragments of the same wavefront edge would re-merge. *P* has, by definition, *h* holes. We transform *P* into a weakly-simple polygon $P^{\prime }$ by adding *h* edges between pairs of vertices of *P*. Each of these edges connects one hole of *P* to the outer boundary of *P*, either directly or indirectly via already connected holes. The body $P^{\prime }\cup T(P,\sigma )$ now has $v^{\prime }=v+n$ vertices, $e^{\prime }=e+n+h$ edges, and $f^{\prime }=n+1$ faces. We can triangulate each (weakly-simple) face without changing the Euler–Poincaré characteristic. Thus, by applying the formula of Euler–Poincaré to the triangulated surface, we obtain e=n+v−1+2g−h.  □

Corollary 17 *Let P be a polygon with h holes, and let σ be an assignment of positive weights to the edges of P. Then*

e=n+v−1+h,

*where v and e denote the number of straight-skeleton nodes and arcs.*

Proof By Lemma 4, T(P,σ) is a terrain that projects to *P*. Hence the genus *g* of the body enclosed by P∪T(P,σ) is equal to *h*. Thus, Lemma 16 implies that e=n+v−1+h.  □

Lemma 18 *Let P be a simple polygon. Then* S(P,σ) *is a tree if and only if the body enclosed by* P∪T(P,σ) *has genus zero.*

Proof Let *v* and *e* denote the number of straight-skeleton nodes and arcs and let *g* denote the genus of the body enclosed by P∪T(P,σ). Since *P* is simple, S(P,σ) is connected by Corollary 14. S(P,σ) is a tree if and only if e=n+v−1. Lemma 16 says that e=n+v−1+2g. Thus S(P,σ) is a tree if and only if g=0.  □

Corollary 19 *Let P be a simple polygon and let* σ(e)>0 *for all wavefront edges e. Then* S(P,σ) *is a tree.*

### 5.5 Convex polygons

In the following section we investigate the weighted straight skeleton and roof-model of a strictly convex polygon *P*, i.e., a polygon whose vertices have interior angles less than *π*.

Lemma 20 *There exists a strictly convex polygon P with weights chosen from the set* {−3,−1,3} *such that* S(P,σ) *has crossings.*

**Fig. 11:**
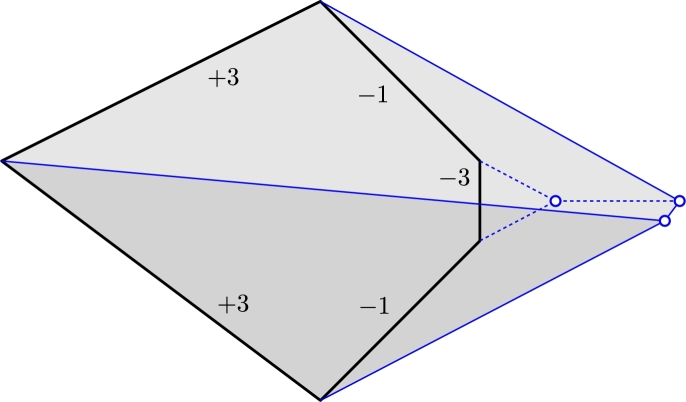
Even if *P* is a convex polygon, S(P,σ) may have crossings. The two left-most edges have weight +3, the vertical edge has weight −3, the two remaining edges have weight −1.

Proof The polygon is shown in Fig. 11.  □

Lemma 21 *Let P be a strictly convex polygon, and let σ be an assignment of arbitrary weights to its edges. Then the wavefront* $W_{P,\sigma }(t)$ *is strictly convex at all times* t≥0 *.*

Proof We first note that the angles between neighboring edges of the wavefront stay constant between events. The initial wavefront is strictly convex as *P* is convex. By induction over all events (i) every event is an edge event and (ii) the wavefront remains strictly convex after each event.  □

The following lemma is given as Lemma 6.1 in [13].

Lemma 22 *A polyhedron is convex if and only if its edges are convex.*

Lemma 23 *A polyhedron* P *is convex if and only if there is a plane H such that*

(i) *all intersections of* P *with planes parallel to H are convex, and*
(ii) *all edges of* P *parallel to H are convex.*

Proof If P is convex then (i) and (ii) are trivial. For the other direction, by Lemma 22 it suffices to show that those edges *e* that are not parallel to *H* are convex. We choose a point *p* in the interior of *e* and a parallel plane $H^{\prime }$ of *H* that contains *p*. The intersection $H^{\prime }\cap P$ gives a convex polygon by (i) and *p* is a convex vertex of $H^{\prime }\cap P$. Thus, the edge *e* is convex.  □

Theorem 24 *Let P be a strictly convex polygon, and let σ be an assignment of arbitrary weights to the edges. Then the body enclosed by* P∪T(P,σ) *is a convex polyhedron.*

Proof Lemma 21 tells us that the wavefront of a strictly convex polygon is always strictly convex too. Thus, Lemma 23 (i) trivially holds for *H* being the *xy*-plane and it remains to check Lemma 23 (ii). Assume that *e* is an edge in the roof that is horizontal, i.e., parallel to the *xy*-plane. As the wavefront forms a strictly convex polygon at all times, the horizontal roof edge *e* must have been created when two parallel wavefront edges meet and, thereby, cause the strictly convex wavefront polygon to collapse. In particular, this implies that *e* is a ridge and, hence, is convex in the roof, satisfying Lemma 23 (ii). Thus, P∪T(P,σ) is a convex polyhedron by Lemma 23.  □

Every straight-skeleton face corresponds to a face of the roof. By Theorem 24, the straight-skeleton faces are not just monotone but in fact convex:

Corollary 25 *Let P be a strictly convex polygon, and let σ be an assignment of arbitrary weights to its edges. Then the straight-skeleton faces are convex.*

We can even give a characterization of the body B enclosed by the roof model. Each face of B is either a face of the roof model (and hence uniquely corresponds to a face f(e) of the straight-skeleton and hence an edge *e* of *P*), or it is *P*. For an edge *e* of *P*, let $H_{\sigma }(e)\subset R^{3}$ be the half-space that supports the face of T(P,σ) that corresponds to f(e) and that has B inside. Let $H(P):=R^{2}\times [0,\infty )$; this half-space supports *P* and looks “upward”, hence also contains B. Using E(P) to denote the edges of *P*, Theorem 24 then implies:

Corollary 26 *Let P be a strictly convex polygon, and let σ be an assignment of arbitrary weights to the edges. Then the body* B *enclosed by* P∪T(P,σ) *satisfies*

$$B=H(P)\cap \underset{e\in E(P)}{⋂}H_{\sigma }(e).$$

A long standing problem of ordinary straight skeletons was to find non-procedural characterizations [3], [14]. Corollary 26 allows us to characterize any weighted straight skeleton of a convex polygon as the projection of all edges of the polyhedron $H(P)\cap {⋂}_{e\in E(P)}H_{\sigma }(e)$ to the plane $R^{2}\times \{0\}$. Note that $H_{\sigma }(e)$ can easily be determined from *e* and σ(e). Since all half-spaces (except for H(P)) correspond to edges of *P* and, thus, occur in sorted circular order along *P*, the results of Aggarwal et al. [15] on computing certain kinds of convex hulls in three dimensions are applicable, thus establishing the proof of the following theorem.

Theorem 27 *Let P be a strictly convex n-gon, and let σ be an assignment of arbitrary weights to its edges. Then* S(P,σ) *can be computed in* O(n) *time.*

We also want to remark that the restriction to $H(P)=R^{2}\times [0,\infty )$ is only a technicality in the following sense: If we were to extend the definition of S(P,σ) and T(P,σ) to negative times, then $T(P,\sigma )={⋂}_{e\in E(P)}H_{\sigma }(e)$.

Lemma 28 *Let P be a strictly convex polygon, and let σ be an assignment of arbitrary weights to the edges. Then* S(P,σ) *is a tree.*

Proof By Theorem 24, the roof has genus zero. By Lemma 18, S(P,σ) is a tree. (Recall that we insert an infinite straight-skeleton node for the wavefront polygon should it propagate towards infinity.)  □

Corollary 29 *Let P be a strictly convex polygon. For any weight function σ, the straight skeleton* S(P,σ) *has* n+v−1 *arcs, where n is the number of vertices of P and v is the number of nodes in* S(P,σ) *.*

## 6 Conclusion

In this paper, we studied weighted straight skeletons. We showed that the natural definition already comes with ambiguities and that many properties that seemed to be natural for unweighted straight skeletons do not remain valid for weighted straight skeletons. Hence, caution must be used when applying weighted straight skeletons. A general observation is that if weights are positive or the input polygon is convex then most, albeit not all, properties of straight skeletons carry over to the weighted counterpart.

In some applications of weighted straight skeletons, the weights are chosen in a specific way. In Haunert and Sester [6] the weights are positive and, as we showed in this paper, the weighted straight skeleton is a crossing-free tree, as required by the application. In Barequet et al. [5], however, weighted straight skeletons of polygons are considered, where negative weights in general cannot be avoided.^5^ Still the weights are chosen in some specific way such that the weighted straight skeleton may have the desired properties. Showing this remains as an open problem.

For convex polygons we were able to characterize the roof and, thus, the straight skeleton as an intersection of half-spaces. Cheng and Vigneron [16] and Huber and Held [14] used the motorcycle graph and the generalized motorcycle graph, respectively, to achieve a similar characterization for ordinary straight skeletons by means of the lower envelope of partially defined linear functions. Generalizing this idea to weighted straight skeletons of non-convex polygons or polygons with holes is an important task and remains open. Similarly, Cheng and Vigneron [16] and Huber and Held [14] used the (generalized) motorcycle graph to compute the straight skeleton of non-degenerate polygons and arbitrary planar straight-line graphs. Can these algorithms be generalized to weighted straight skeletons? This open problem is directly related to characterizing weighted straight skeletons by means of lower envelopes.

In a recent paper [17], we showed how to characterize polygonal tessellations *S* of the plane to ascertain which form the straight skeleton of some planar straight-line graph *G* and how to determine *G* from its putative straight skeleton *S*. Can those methods be generalized to weighted straight skeletons?

In this paper, we discussed the weighted straight skeleton of polygons and polygons with holes. A generalization of straight skeletons to a non-intersecting collection of polygons is obvious. One may also generalize the weighted straight skeleton to planar straight-line graphs *G*. For the ordinary straight skeleton, every edge of *G* sends out a wavefront edge on either side and every terminal vertex of *G* sends out its own wavefront edge. Hence, it may make sense to restrict the weights to be positive, but the weights of wavefront edges emanating from the same edge of *G* need not be equal.

Aichholzer et al. [18] used a generalization of straight skeletons where wavefront edges do not necessarily start to propagate at time zero. Such straight skeletons could be called additively-weighted straight skeletons [3], in contrast to multiplicatively-weighted straight skeletons, which are studied in this paper. Systematically investigating additively-weighted straight skeletons remains as future work.

## Footnotes

1 One could also argue for omitting ghost vertices, thus effectively merging *e* and $e^{\prime }$ into one edge of the wavefront. But then, even in the unweighted case, the straight skeleton is not always connected.

2 One could expand the definition to zero weights, resulting in stationary wavefront edges. The roof model is then still well-defined, and the straight skeleton could be considered to be the projection of the edge graph of the roof model onto the ground plane. However, straight-skeleton arcs may then degenerate into points, and faces of edges may be line segments. We forbid zero-weight edges here to avoid such degeneracies.

3 A simple polygon *P* can be characterized by its boundary *B*, which is a closed Jordan curve consisting of straight-line segments. In a weakly-simple polygon, boundary segments may overlap. We still require that P∖B is connected and simple, however.

4 Note that adding ghost vertices when fragments of the same edge re-combine is crucial here, otherwise no arc would emanate from the created skeleton node.

5 The weighted straight skeleton is used to determine the wavefront topology of three-dimensional polyhedra P. When P is a pyramid with a very thin U-shaped base then determining the three-dimensional initial wavefront of the top vertex necessarily involves negative weights.
